# Mesenchymal Stem Cells in Acquired Aplastic Anemia: The Spectrum from Basic to Clinical Utility

**DOI:** 10.3390/ijms24054464

**Published:** 2023-02-24

**Authors:** Xing-An Wang, Ju-Pi Li, Kang-Hsi Wu, Shun-Fa Yang, Yu-Hua Chao

**Affiliations:** 1 Department of Pediatrics, Chung Shan Medical University Hospital, Taichung 402, Taiwan; 2 School of Medicine, Chung Shan Medical University, Taichung 402, Taiwan; 3 Institute of Medicine, Chung Shan Medical University, Taichung 402, Taiwan; 4 Department of Medical Research, Chung Shan Medical University Hospital, Taichung 402, Taiwan; 5 Department of Clinical Pathology, Chung Shan Medical University Hospital, Taichung 402, Taiwan

**Keywords:** aplastic anemia, bone marrow failure, cell therapy, mesenchymal stem cells

## Abstract

Aplastic anemia (AA), a rare but potentially life-threatening disease, is a paradigm of bone marrow failure syndromes characterized by pancytopenia in the peripheral blood and hypocellularity in the bone marrow. The pathophysiology of acquired idiopathic AA is quite complex. Mesenchymal stem cells (MSCs), an important component of the bone marrow, are crucial in providing the specialized microenvironment for hematopoiesis. MSC dysfunction may result in an insufficient bone marrow and may be associated with the development of AA. In this comprehensive review, we summarized the current understanding about the involvement of MSCs in the pathogenesis of acquired idiopathic AA, along with the clinical application of MSCs for patients with the disease. The pathophysiology of AA, the major properties of MSCs, and results of MSC therapy in preclinical animal models of AA are also described. Several important issues regarding the clinical use of MSCs are discussed finally. With evolving knowledge from basic studies and clinical applications, we anticipate that more patients with the disease can benefit from the therapeutic effects of MSCs in the near future.

## 1. Introduction

Aplastic anemia (AA) is a paradigm of bone marrow failure syndromes characterized by peripheral pancytopenia and bone marrow hypoplasia. The first formal description dates back to 1888 when Paul Ehrlich reported a young pregnant women with profound anemia, bleeding, high fever, and eventually death [1]. AA can be graded by the severity of cytopenias in the peripheral blood [2]. The criteria of severe aplastic anemia (SAA) are bone marrow hypocellularity of less than 25% and at least two of following: absolute neutrophil count < 0.5 × 10^9^/L, platelet count < 20 × 10^9^/L, and reticulocyte count < 20 × 10^9^/L. In very severe AA, there is extreme neutropenia of less than 0.2 × 10^9^/L. In nonsevere AA, hypocellular bone marrow is noted but peripheral blood values do not meet the criteria of SAA. SAA is potentially life-threatening if dedicated treatment is not implemented.

Acquired AA can affect patients of all ages, with an annual incidence of about 2 per million population [3]. A variety of insults, such as drugs, chemicals or irradiation, and infections, can lead to the impairment of hematopoiesis, which is the main feature of AA. A specific cause cannot be identified in a large proportion of patients and is termed “idiopathic AA”. Historically, immunosuppressive therapy (IST) and hematopoietic stem cell transplantation (HSCT) have been the mainstay of treatment for these patients. Having unique properties, the clinical application of mesenchymal stem cells (MSCs) is evolving rapidly in the recent decade for many human diseases, including AA.

Here, we present a comprehensive review of current concepts regarding the insufficiency of MSCs in acquired idiopathic AA and the clinical use of MSCs in patients with the disease. We also summarized the pathophysiology of AA, the properties of MSCs, and preclinical results of MSC therapy in animal models of AA. In view of different entities, a discussion about congenital AA with an identified germline mutation is outside the scope of this review.

## 2. Pathophysiology of Aplastic Anemia

**Figure 1:**
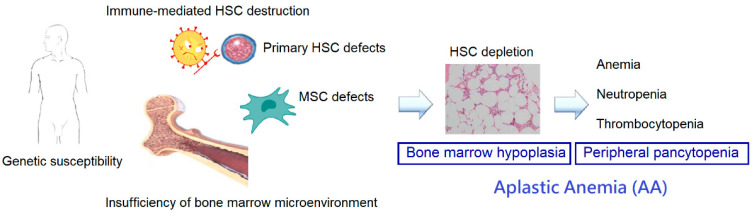
Pathophysiology of acquired idiopathic aplastic anemia.

AA is characterized by pancytopenia in the peripheral blood and hypocellularity in the bone marrow. When AA is suspected, a comprehensive evaluation should be performed to exclude other mimicking conditions and search for underlying etiologies. Despite of these efforts, a specific cause cannot be identified in a large proportion of patients and is termed “idiopathic”. The pathogenesis of acquired idiopathic AA is complex, and we summarized the current concepts in the following sections (Figure 1).

### 2.1. Immune Dysfunction

The immune-mediated destruction of hematopoietic stem cells (HSCs) is the most widely accepted mechanism of hematopoietic failure in AA. Available evidence suggests the gross homeostatic dysregulation of the T cell repertoire in AA, and T cell attack on marrow cells of AA has been illustrated in vitro and in vivo. It has been demonstrated that the percentage of activated CD8+ cytotoxic T cells is increased in the both bone marrow and peripheral blood of patients with AA. In vitro coculture with CD8+ T cells isolated from patients with AA was found to inhibit colony formation and enhance apoptosis of CD34+ cells isolated from normal individuals [4,5,6], suggesting CD8+ cytotoxic T cell dysfunction in AA. Abnormalities in the number and function of CD4+ T cells have also been documented in AA, with increased T helper (Th)1, Th2, and Th17 cells [7]. Regulatory T cells (Tregs) are believed to control autoimmunity by suppressing autoreactive T cells and play an important role in the maintenance of immune homeostasis. A decrease in the number of CD4+ CD25+ FOXP3+ Tregs was found in almost all patients with AA [8], and there was an inverse relationship between the numbers of Th17 cells and Tregs in the peripheral blood of patients [9]. Meanwhile, hyperactive T cells in AA may release a variety of inflammatory cytokines, such as interferon-γ (IFN-γ), tumor necrosis factor-α (TNF-α), and interleukin (IL)-17, thus with elevated concentrations in the serum and bone marrow plasma [10,11,12]. These cytokines were found to confer additional hematopoietic suppression by increasing Fas expression on CD34+ progenitor cells and inducing the programmed cell death of these cells [6]. On the other hand, the effectiveness of IST in the treatment of AA provides compelling clinical evidence for the immune-mediated nature of the disease.

### 2.2. Deficiencies of HSCs

HSCs are capable of self-renewal and differentiation into various hematopoietic cells and are critical for the maintenance of the hematopoietic system. There is evidence of primary deficiencies, both quantitative and qualitative, in AA HSCs. Patients with AA have decreased numbers of HSCs at diagnosis [13,14], and these cells were found to display poor plating efficiency for colony formation [15,16]. Increased apoptosis of CD34+ cells in both the bone marrow and peripheral blood was observed in patients with AA [17,18]. Some intrinsic abnormalities predispose AA HSCs to apoptosis, resulting in HSC depletion. The upregulated expression of Fas antigen, which is a receptor molecule in the death signaling pathway, was frequently found on CD34+ cells in AA [19]. Abnormal expression of the apoptotic modulators Bcl-2 and Bcl-x was associated with the increased apoptosis of CD34+ cells in AA [20]. In addition, downregulated expression of cell cycle check point genes, such as *CDK6*, *CDK2*, *MYB*, *MYC*, and *FANCG*, was found in AA HSCs [21] and may compromise their replicative ability. Meanwhile, about one-third of patients with AA have telomere attrition in their leukocytes [22], and the telomere length at diagnosis correlates with disease severity and clinical outcomes [23,24]. Only a small subset of patients harbors mutations in the telomerase complex genes, such as *TERT* or *TERC* [25,26], and the majority of patients do not have these identifiable mutations. Therefore, multiple mechanisms may contribute to telomeric shortening in AA, including increased stem cell turnover, unidentified damage to telomeres, and genetic defects.

### 2.3. Genetic Susceptibility

Genetic factors play an important role in the pathogenesis of AA. Because patients harboring cytogenetic abnormalities may exhibit distinct pathogenesis and clinical manifestations, these patients should be discussed separately.

Several studies have reported the association between certain human leukocyte antigen (HLA) alleles and the predisposition of AA. AA is more common in HLA-DR2-positive individuals than in the general population and in particular with the HLA-DRB1*15 allele in Asia. Moreover, DRB1*03, DQB1*0601, and DQB1*0603 were found to be either susceptible or protective alleles [27,28]. Although still being elucidated, HLA allelic variations may contribute to the activation of autoreactive T cells and the protective failure of Tregs.

Recent advances in genomic analysis have revealed the complexity of somatic mutations and clonal hematopoiesis in AA. *PIGA* mutations were frequently detected in patients with AA at diagnosis but without paroxysmal nocturnal hemoglobinuria-related symptoms [29]. About one third of patients have somatic mutations in myeloid cancer candidate genes and in a limited set of genes, such as *DNMT3A*, *ASXL1*, and *BCOR*, and at a lower variant allele frequency [30]. The prevalence of these mutations increases with age, with a lower incidence in pediatric population. Some mutations were related to clinical outcomes; some may have potential to evolve from AA to myelodysplastic syndrome [31].

### 2.4. Alterations in the Bone Marrow Microenvironment

As known, bone marrow provides the distinct microenvironment for hematopoiesis. The cellular elements, including endosteal, vascular, and perivascular cells, were found to be markedly decreased in the bone marrow of patients with AA [32], implicating the possibility of microenvironmental impairment in the bone marrow. MSCs play a central role in the establishment of the bone marrow niche, and their defects may lead to the development of AA. In the following sections, the characteristics of MSCs and the alterations of bone marrow MSCs in patients with AA are discussed in detail.

## 3. Mesenchymal Stem Cells

### 3.1. MSCs in the Bone Marrow

There are three main cellular systems in the bone marrow: hematopoietic, endothelial, and stromal [33]. The stromal cell system, which was first proposed by Owen et al. in 1985 [34], is composed of a variety of nonhematopoietic stromal cells of a mesenchymal origin. MSCs, an important component of the stromal bone marrow, constitute only a small percentage of marrow cells, about one in 3.4 *×* 10^4^ [35]. Being the so-called stem cells, MSCs within the bone marrow have to maintain a level of self-renewal and give rise to various mesenchyme-lineage cells [36].

In the bone marrow, MSC-derived stromal cells provide the specific microenvironment for hematopoiesis by establishing an appropriate scaffold of extracellular matrix molecules along with a complex network of paracrine factors. MSCs secrete a number of cytokines, which can act not only on hematopoietic cells but also on stromal cells [37]. Numerous growth factors, such as stem cell factor, granulocyte macrophage-colony stimulating factor, granulocyte colony-stimulating factor, and macrophage colony-stimulating factor, have positive effects on hematopoiesis. MSCs also produce negative regulators of hematopoiesis, such as IL-8, macrophage inflammatory protein-1, and transforming growth factor-β (TGF-β). In addition, MSCs involve cell migration and homing to the bone marrow via the expression of adhesion molecules, including integrins, intercellular adhesion molecule-1, vascular cell adhesion molecule-1 (VCAM-1), and other molecules of the extracellular matrix [38,39]. Given the significant role in the hematopoietic niche, many studies have demonstrated the promotive effects of MSCs on in vitro HSC expansion [40,41,42,43]. In animal models, the infusion of MSCs has been shown to enhance the engraftment of donor HSCs after transplantation [43,44,45].

### 3.2. Properties of MSCs

**Figure 2:**
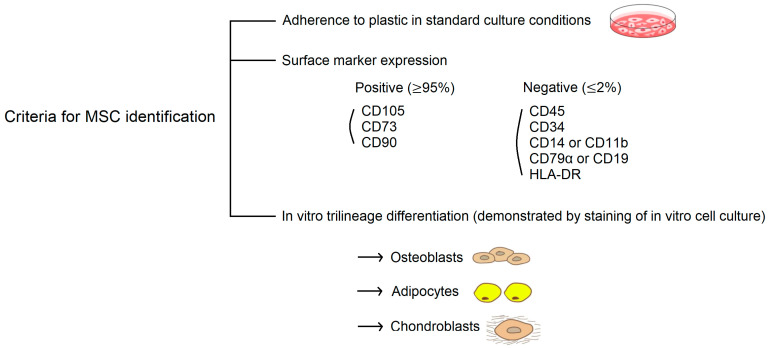
Criteria of the International Society for Cellular Therapy (ISCT) for the definition of human MSCs.

According to the consensus statement by the International Society for Cellular Therapy (ISCT), human MSCs are defined by their in vitro growth pattern, the expression of specific surface markers, and the multipotent differentiation potential (Figure 2) [46]. Having a spindle-shaped fibroblastic morphology, MSCs must be plastic-adherent when maintained in standard culture conditions. MSCs must express CD105, CD73, and CD90 and lack expression of CD45, CD34, CD14 or CD11b, CD79α or CD19, and HLA-DR. With a broad differentiation potential, the most unique property to identify MSCs is the capacity for trilineage mesenchymal differentiation, including osteoblasts, adipocytes, and chondroblasts.

MSCs express HLA-class I molecules, but not class II. MSCs do not express costimulatory molecules CD80 (B7-1), CD86 (B7-2), CD40, and CD40 ligand and probably, therefore, do not activate alloreactive T cells [47,48,49]. In vivo, transplanted allogeneic MSCs can be detected in recipients at extended time points, implicating the lack of immune recognition and clearance [50]. In humans, numerous clinical reports have demonstrated that infused mismatched allogeneic MSCs do not trigger vigorous T cell responses in recipients without the risk of transferred cell rejection [51,52,53]. These data offer evidence that MSCs are immunologically inert or potentially tolerogenic, indicating the convenience for their clinical utility.

### 3.3. Immunomodulation by MSCs

**Figure 3:**
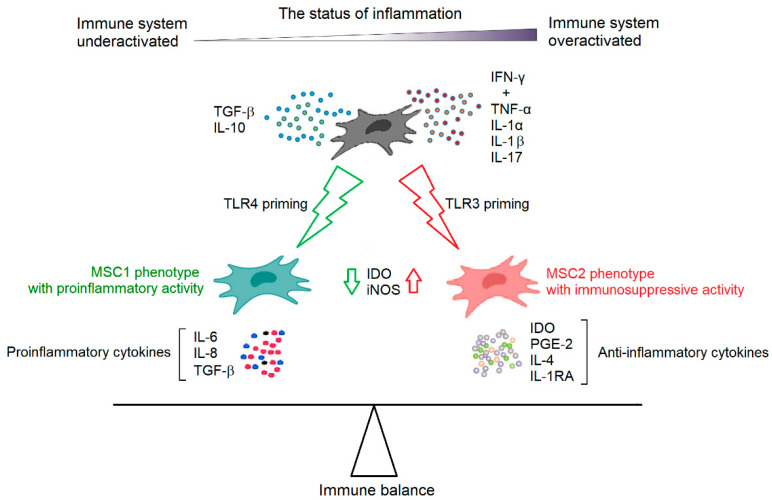
Plasticity of MSCs in immunomodulation. Being receptive to environmental signals, MSCs can gain immunomodulatory capacity for both promoting and inhibiting immune reactions. IDO and/or iNOS serve as an “on-off” switch that determines the outcome of immunomodulation by MSCs.

MSCs are able to regulate the activities of immune cells from both innate and adaptive immune systems, but their immunomodulatory capacity is not constitutive. MSCs require “licensing” to gain their immunosuppressive function after stimulation from certain inflammatory cytokines and are therefore chemokine-dependent. IFN-γ is critical for the chemokine induction in MSCs [54,55,56]. However, the optimal induction of immunosuppression requires the concurrent addition of other proinflammatory cytokines, such as TNF-α, IL-1α, IL-1β, and IL-17 [55,56,57]. Constitutively, MSCs express low levels of immunomodulatory molecules, including indoleamine 2,3-dioxygenase (IDO), prostaglandin E-2 (PGE-2), TGF-β, and hepatocyte growth factor (HGF). These inflammatory cytokines can differentially regulate their expression under inflammatory conditions [58]. Again, IFN-γ in particular plays an important role in regulating MSC immunomodulatory factor expression [58]. On the contrary, immunosuppressive cytokines, such as TGF-β and IL-10, are believed to serve a counterbalancing role by inducing less immunosuppressive MSCs and thus become immune-enhancing. It was observed that TGF-β abolishes the immunosuppressive capacity of MSCs by downregulating the expression of IDO or inducible NO synthase (iNOS) in MSCs [59]. In addition to cytokines, Toll-like receptor (TLR) signaling has also been implicated in the licensing of MSCs, and the stimulation of specific TLRs on MSCs may greatly affect their immune-modulating responses. TLR4 priming can induce a proinflammatory signature of MSCs (MSC1 phenotype), which mostly involves inflammatory mediators, such as IL-6, IL-8, and TGF-β. In contrast, TLR3-primed MSCs represent immunosuppressive activities (MSC2 phenotype) and secrete IDO, PGE-2, IL-4, and IL-1RA [60]. These data indicate that the inflammatory status in their microenvironment determines the immunoregulatory fate of MSCs (Figure 3). They are rendered immunosuppressive in the presence of strong inflammation, but week inflammation may cause MSCs to promote immune responses [61].

Having profound immunomodulatory potential, MSCs are receptive to environmental cues and orchestrate the activation, proliferation, and function of immune cells from both innate and adaptive immune systems. Under inflammatory conditions, the immunosuppressive activities of MSCs have been demonstrated to prevent overstimulation of the immune system. Monocyte/macrophage modulation is critical in the MSC-mediated immunomodulatory process. MSCs can inhibit monocyte differentiation from CD34+ HSCs and promote macrophage polarization into an anti-inflammatory M2 phenotype [62,63]. MSCs can affect the differentiation of dendritic cells (DCs) from monocyte precursors [64,65,66]. They could inhibit DC maturation and activation by downregulating the expression of presentation molecules (HLA-DR and CD1a) and costimulatory molecules (CD80 and CD86) in DCs [67,68]. MSCs also exert immunosuppressive effects on mature DCs by inhibiting their migration, compromising their antigen-presenting abilities, repressing their stimulation of lymphocyte proliferation, and decreasing their secretion of inflammatory mediators [69]. Moreover, MSCs can modulate the immune responses of natural killer cells by downregulating their proliferation, cytokine production, and cytolytic activity [70]. MSCs exert immunomodulatory effects on these innate cells mainly through paracrine mechanisms, involving a complicated network of cytokines.

MSCs act on the adaptive immune system in multiple ways, through both direct cell contacts and paracrine effects. MSCs are able to inhibit the proliferation, differentiation, chemotaxis, and immunoglobulin production of B cells [71,72,73,74]. MSCs can also promote the induction of IL-10-producing regulatory B cells, which restrain inflammation by enhancing the conversion of effector CD4+ T cells into Tregs [75]. MSCs influence the behavior of T cells significantly, including proliferation, activation, cytokine secretion, and cytotoxicity [76]. Under inflammatory conditions, MSCs inhibit the expression of Th1 proinflammatory cytokines and enhance Th2 factors, resulting in a more tolerant immune status [50]. MSCs can also regulate the balance between Th1 and Th2 cells through interactions with DCs and natural killer cells [77]. An important mechanism of MSC-mediated immunomodulation of T cells is the generation of Tregs, which are essential for immune homeostasis and the prevention of alloreactive and autoimmune diseases [78]. MSCs can directly induce Treg differentiation through the TLR–Notch pathway and the secretion of TGF-β, IDO, and iNOS [79,80,81]. By increasing IL-10 secretion and decreasing IFN-γ and IL-17 production, MSCs can also promote Treg differentiation from CD4+ T cells and suppress Th1 and Th17 differentiation [82]. MSCs play a role in modulating the balance between Tregs and Th17 cells, and the Treg/Th17 ratio is implicated in shaping the outcome of immune responses (immunosuppression versus inflammation) [83,84]. Due to their great immunomodulatory functions, MSC therapy has been used in numerous immune- or inflammation-associated disorders in humans.

### 3.4. MSCs from Various Origins

MSCs are derived from mesodermal progenitor cells, and they were firstly identified in the bone marrow [85]. In addition to bone marrow, MSCs can also be isolated from a broad spectrum of tissues, including adult tissues (peripheral blood, fat, and dental pulp) and fetal tissues (umbilical cord, cord blood, placenta, amniotic membrane, and amniotic fluid). The frequency of MSCs in different tissues varies greatly, and their ontological origin also has considerable influence on their performance. The umbilical cord is much richer in MSCs than cord blood [86]; adult bone marrow is a reliable source, but peripheral blood is not [35]. There are several differences between fetal-type MSCs and adult-type MSCs. Fetal-type MSCs appear to have greater expansion capacity, which may be due to their longer telomeres, greater telomerase activity, and higher expression of telomerase reverse transcriptase [87,88,89]. Fetal-type MSCs are less lineage-committed and display lower levels of HLA-class I [90]. Fetal-type MSCs and adult-type MSCs may express different cytokine profiles, immunomodulatory properties, and differentiation potential [88,89,90,91,92].

## 4. Alterations to MSCs in AA

### 4.1. Hematopoietic Support

The regulation of hematopoiesis depends on the interaction between HSCs and various cells within the bone marrow niche. Several studies have demonstrated the involvement of MSCs in the functional restriction of HSCs in AA. Using a long-term culture system, bone marrow stromal cells of patients with AA were found to be insufficient to maintain normal HSCs and failed in confluent growth [93,94]. When cocultured with AA MSCs, the proliferation of peripheral blood mononuclear cells and the colony-forming capacity of CD34+ cells were significantly reduced [95,96]. These data implicated the insufficiency of AA MSCs in hematopoietic support. Downregulated expression of *VCAM-1* in AA MSCs was proposed as being associated with the impairment of in vitro growth support and in vivo engraftment of CD34+ cells [97]. However, multiple intrinsic defects in the bone marrow MSCs of patients with AA may exist.

### 4.2. Proliferative Potential

Impaired proliferative potential is the hallmark of MSCs in AA. Using population doubling as the indicator, we firstly reported that AA MSCs had a worse average population doubling at each passage and a smaller cumulative population doubling from passage 4 to 6 [98]. We observed that three of five AA MSC cultures stopped proliferating at passage 5, implicating the possibility of early senescence in AA MSCs [95]. We further determined the distribution of cultured cells in different cell cycle phases by flow cytometry and found a high percentage of cells in the abnormal sub-G1 phase in AA MSCs [95]. Our data suggested that an increased apoptotic rate of AA MSCs may contribute to their poor proliferative capacity and early senescence. Our findings were confirmed by numerous studies thereafter [96,99,100,101,102,103]. The worse proliferative potential of AA MSCs was documented based on different parameters, including population doublings [96,99,100], a CCK-8 assay [100,101], growth curves [102], and colony-forming potential [103]. Higher rates of apoptotic cells in AA MSCs were measured via cell cycle analysis [100] and an annexin V-affinity assay [101,103]. AA MSCs were found to be prone to senescence, shown by a higher proportion of β-galactosidase-stained cells [100] and failure to passage [102].

### 4.3. Surface Marker Expression

Phenotypic characterization is an important ISCT criterion to define MSCs, as shown in Figure 2 [46]. Flow cytometric analysis has been used to examine the alteration in surface marker expression in AA MSCs. The study by Bueno et al. was the only one that fully complied with all the marker criteria established by the ISCT [104], and most studies used six to eight markers as the indicator. Consistent results showed that no difference was noted in the expression of any single surface marker between MSCs derived from patients with AA and controls [105].

### 4.4. Differentiation Capacity

The final criterion of the ISCT for the definition of MSCs is their in vitro trilineage mesenchymal differentiation capacity (Figure 2) [46]. We firstly reported lower osteogenic and adipogenic capacities of AA MSCs following differentiation induction [98]. Thereafter, a variety of qualitative and quantitative modalities was used to assess alterations to AA MSCs in differentiation tendencies towards osteoblasts, adipocytes, and chondroblasts, including staining methods, RT-qPCR, and Western blot analysis. Lower osteogenic and chondrogenic differentiation propensities of AA MSCs were confirmed in most studies. However, there was an apparent discrepancy in the results of adipogenesis in AA MSCs, which included increased adipogenic differentiation potency in some studies and decreased in others [105]. The discrepancy can be explained by the complex pathogenesis of idiopathic AA and heterogeneous study populations. Nevertheless, alterations in the differentiation potential of AA MSCs may exist.

### 4.5. Immunomodulation

Immune-mediated HSC destruction is an important pathogenic mechanism of idiopathic AA, and MSCs have great immunomodulatory functions. The dysregulation of immune cells from MSCs may impact immune homeostasis in the bone marrow microenvironment. Our previous study demonstrated aberrant cytokine profiles in the conditioned medium of AA MSCs with increased concentrations of IL-1β, IL-6, IFN-γ, and TNF-α, suggesting the association between aberrant paracrine factors secreted by MSCs and the hyperinflammatory marrow niche in AA [95]. Bacigulupo et al. observed deficiency in the ability of AA MSCs to downregulate T cell priming, proliferation, and cytokine release [106]. Hu et al. demonstrated impaired inhibition of AA MSCs based on the activation of CD4+ and CD8+ T cells and differentiation towards Th1 and Th17 cells [100]. Li et al. found that AA MSCs were reduced in suppressing the proliferation and clonogenic potential of CD4+ T cells and the production of IFN-γ and TNF-α by CD4+ T cells [107]. We recently examined the influence of AA MSCs on the differentiation of CD4+ T cells into CD4+CD25+FoxP3+ Tregs under Treg polarization conditions. AA MSCs showed promotive effects on Treg differentiation, but there were inconsistent changes based on TGF-β and IL-1β levels in the supernatant, implying that a large number of dysfunctional Tregs was produced when CD4+ T cells were cocultured with AA MSCs during differentiation [108]. Collectively, these data provide evidence for the disordered immunomodulatory function of MSCs in AA.

### 4.6. Gene Expression

Consistent with the anomalous biological performance of bone marrow MSCs in AA, differential gene expression profiles were reported in the literature. Using microarray analysis, we found two different gene expression patterns of MSCs in children with AA, suggesting the heterogeneity of idiopathic AA. Fourteen genes associated with DNA synthesis and growth factors, including POLE2, HFG, KIF20A, TK1, IL18R1, KITLG, FGF18, RRM2, TTK, CXCL12, DLG7, TOP2A, NUF2, and TYMS, were markedly downregulated in AA MSCs [109]. Li et al. performed a comprehensive analysis and confirmed the consistency between the abnormal biological features and the alteration of gene expression profiles in AA MSCs. They observed decreased expression in a number of genes implicated in the cell cycle, cell division, proliferation, chemotaxis, and hematopoietic cell lineage in AA MSCs. Conversely, the expression of genes related to apoptosis, adipogenesis, and the immune response was increased in AA MSCs [103]. With the advance in experimental tools, Hue et al. documented the differential gene expression pattern of AA MSCs using genome-wide RNA sequencing. They found that the differentially expressed genes were principally associated with immunoregulation, cell cycle, and cell division [100].

Aside from the global view of gene expression profiles, there were several reports about the differential expression of an individual gene, which involved one or more defective properties of MSCs in AA. Regarding the contribution of MSCs to angiogenesis, downregulated expression of VCAM-1 and ANG-1 genes was noted in AA MSCs [97,110]. Jiang et al. found downregulated FGF2 expression in AA MSCs, suggesting their compromised ability of self-renewal and impaired support of HSCs [102]. As an important molecule for the maintenance of HSCs in the bone marrow, decreased CXCL12 expression in AA MSCs was found in our previous study, and the knockdown of CXCL12 compelled control MSCs to behave like AA MSCs [109]. A number of genes was reported to contribute to the alteration in the differentiation potential of AA MSCs, including CBF-α1, RUNX2, and BGLAP in osteogenesis, SOX9 and ACAN in chondrogenesis, and PPAR-γ, ADIPOQ, and FABP4 in adipogenesis [105].

## 5. MSC Therapy in Animal Models of AA

In view of the promotive effects of MSCs on hematopoiesis, various murine models of AA have been used to examine the efficacy of MSC therapy in the context of AA (Table 1). Several attempts were used to induce hypocellular bone marrow in animals, including the administration of toxic or chemical agents, irradiation, and lymph node infusion, but none represented the exact etiology of human idiopathic AA. Although not perfect, lymph node cell infusion combined with irradiation may be superior to irradiation alone to mimic AA in humans because immune-mediated HSC destruction is an important mechanism. The source of MSCs was quite different among studies, being autologous, allogenic, or even xenogeneic. In addition, the dosage of MSCs and time points of MSC administration were variable. Nevertheless, MSC therapy could lead to longer survival, better hematopoietic reconstitution, and faster recovery of peripheral blood cells in most studies [111,112,113,114,115,116,117].

## 6. Clinical Application of MSCs in AA

### 6.1. MSC Therapy in Human Diseases

With the growing biologic interest in MSCs, the clinical application of MSCs has been evolving rapidly in the recent decade. More than 1000 clinical trials based on the use of MSCs were implemented to treat various pathologies, according to the US National Institute of Health-Clinical Trial Database (http://clinicaltrials.gov, accessed on 16 February 2022). Several biological properties of MSCs contribute to their therapeutic effects in cell therapy, such as immunomodulation, tissue repair/regeneration, angiogenesis, and antiapoptotic activity [118]. As expected, there are many reports in the literature regarding outcomes of MSC therapy for various human diseases [52,119]. Presumably, diseases with optimal efficacy fall into two main categories. One is associated with immune dysregulation, such as autoimmune diseases (systemic lupus erythematosus, type 1 diabetes mellitus, rheumatoid arthritis), inflammatory diseases (Crohn’s disease, acute respiratory distress syndrome), and graft-versus-host disease (GVHD) after allogeneic transplantation. The other regards tissue regeneration, such as traumatic injury (spinal cord injury, cerebral infarction, ischemic heart disease, wound repair), degenerative diseases (osteoarthritis, liver cirrhosis), and bronchopulmonary dysplasia.

### 6.2. Current Treatment for Patients with AA

For patients with idiopathic SAA, allogeneic HSCT or IST should be used as the first-line therapy. The choice is determined by numerous factors, such as severity of the disease, age of the patient, donor availability, and available medical facility [120]. For pediatric and young adult patients with newly diagnosed SAA, allogeneic HSCT should be pursued when an HLA-identical sibling donor is available. IST with antithymocyte globulin and cyclosporine is the most common alternative frontline therapy for older adult patients and those without a matched sibling donor.

The major advantages of HSCT over IST in SAA are the significant reduction in the risk of relapse and the abrogation of the risk for clonal evolution [121]. However, the risk of graft failure and GVHD after HSCT remains the main challenges in the context of SAA. Graft failure was noted in 11–32% of patients with SAA receiving bone marrow transplantation, and acute severe GVHD and symptomatic chronic GVHD occurred in 11–40% and 21–32%, respectively [122]. Moreover, HSCT using grafts from donors other than HLA-identical siblings, such as matched unrelated and haploidentical donors, offers an option for those who have failed previous IST and lack a matched sibling donor. However, the risk of graft failure and GVHD may significantly increase. Novel strategies are needed to improve the transplant-related mortality and morbidity.

Chemotherapy and radiotherapy prior to HSCT cause bone marrow stromal damage, which may be associated with engraftment delay. GVHD is a paradigm of immune dysfunction. As mentioned above, MSCs can differentiate into various mesenchyme-lineage cells and produce a complex network of paracrine factors [37]. Along with their great immunomodulatory function, MSCs are crucial for maintaining immune homeostasis and repairing tissue damage in the bone marrow. A large body of evidence indicated that MSCs are efficient in the promotion of engraftment, the prevention and treatment of graft failure, and the management of GVHD in HSCT [123]. Furthermore, multiple MSC alterations were found in patients with AA, and these may contribute to bone marrow insufficiency in AA. Taken together, MSC therapy is rational in the context of AA.

With the promising results of MSC therapy using preclinical animal models of AA, MSC transplantation alone or in combination with allogeneic HSCs was performed for patients with AA. The safety and efficacy of MSC therapy were evaluated. As of February 2023, there were nine clinical trials registered on the US National Institute of Health-Clinical Trial Database (http://clinicaltrials.gov, accessed on 16 February 2022) when searching for “MSCs and AA”. Meanwhile, there are many reports in the literature regarding the use of MSCs in AA. In the next paragraphs, the available data on the therapeutic role of allogeneic MSCs for patients with AA are discussed.

#### 6.2.1. MSC Infusion

Five studies regarding allogeneic MSC transplantation for patients with refractory or relapsed AA were reported in the literature (Table 2) [124,125,126,127,128]. Most patients received concomitant immunosuppressants, such as antithymocyte globulin and cyclosporine. Four of the five studies included only a small number of patients. Only one study compared the combination of MSC infusions and IST with IST alone, but the number of patients was quite small [128]. Despite some insufficiency in the quality, all of these reports demonstrated the safety of MSC infusions. However, there were no significant effects on hematopoiesis. Clinical hematologic responses were observed in only a small proportion of patients and were mainly partial. Accordingly, MSC infusions, even multiple times, may not be enough to reconstitute the hematopoiesis in patients with AA.

#### 6.2.2. MSC and HSC Co-Transplantation

In view of the unsatisfactory outcomes of MSC infusion, the co-transplantation of MSCs during HSCT may be a better option for patients with SAA because allogeneic HSCs can result in the direct reconstruction of hematopoiesis in the bone marrow. In addition, MSCs have the potential to overcome the obstacles in HSCT, for example, graft failure and GVHD. We and Jaganathan et al. firstly reported the promotive effects of MSC infusion on hematopoietic engraftment during HSCT in two children and one adult with refractory SAA, respectively [129,130]. No acute or chronic GVHD was found in our patients. Since then, several studies that enrolled a higher number of patients with SAA receiving MSC and HSC co-transplantation were reported (Table 3) [131,132,133,134,135,136,137,138]. Different treatment protocols were used in different studies, and only one retrospective study had a control group [138]. Nevertheless, the great benefit of MSC co-transplantation during HSCT was observed based on enhancing hematopoietic reconstitution, preventing graft rejection, ameliorating GVHD, and improving overall survival in patients with SAA. The efficacy of MSCs appears to be maintained across different patient populations and treatment protocols: the source and cell number of MSCs, the frequency and time point of MSC administration, and the donor and source of HSCs. The safety of MSC co-transplantation was also evaluated, and no immediate infusion or late MSC-associated toxicities were reported. Accordingly, co-transplantation of MSCs during allogeneic HSCT may be superior to traditional HSCT for patients with SAA. Further prospective studies with a larger cohort of patients are needed.

### 6.3. Challenges and Road Ahead

Current clinical results showed the beneficial effects of MSC co-transplantation during HSCT in patients with SAA. Some issues related to the application of MSCs should be further explored, and consensus and guidelines are required to maximize their therapeutic efficacy. Because the number of MSCs obtained from the donor is not enough, passaged cells are used widely in clinical practice. However, the in vitro expansion of MSCs may lead to genetic instability and changes in cell behavior. For example, MSCs may gradually lose their properties of early progenitor cells with passaging, and transforming events may occur [46]. Therefore, conditions for MSC culture should be guaranteed, and it is better to use cells without extensive population doublings. Generally, MSCs within six passages are used in humans [139].

When co-transplanted during HSCT for patients with SAA, MSCs were infused for a single time or multiple times, and the cell doses were 0.5–10 × 10^6^/kg per injection. A single injection with a dose of around 1 × 10^6^/kg before HSC infusion was used most frequently (Table 3). Typically, MSCs are administrated intravenously at doses in 1–2 × 10^6^/kg for most clinical conditions [52]. However, it remains to be verified whether this is the optimal strategy. Tracking studies using labeled MSCs demonstrated that most MSCs were cleared from the body within 24–48 h after infusion. Acting as a hit-and-run attack, multiple injections may be needed in some difficult situations, such as graft rejection and severe GVHD. The injection interval ranged from 3 days to 1 week in most clinical conditions, but the optimal frequency and interval in SAA should be further determined.

The source of MSCs is another important issue. MSCs can be obtained from many kinds of tissues, and their ontological origins may greatly impact their properties and their performance in clinical utility. As we know now, no study has compared head-to-head clinical outcomes of MSC therapy with different origins in a single human disease. Fetal-type MSCs are less lineage-committed and have greater expansion capacity [140]. In contrast, bone marrow MSCs exhibit accelerated senescence significantly with age. In addition, umbilical cords are rich in MSCs, and no invasive procedure is needed to obtain MSCs from umbilical cords. Moreover, umbilical cord-derived MSC products from MSC banks are off-the-shelf, with well-performed characterization and the confirmation of sterility. With the great advantage of availability, umbilical cord-derived MSCs have been used prevalently in many human diseases, including SAA. However, the exact discrepancy in the clinical performance of MSCs from different sources should be evaluated.

Many studies reported that no significant side effects were noted in the clinical use of MSCs. However, MSC administration is not completely free of risks and most patients were monitored only for a short period of time. While a post-infusion febrile reaction was reported as the most frequent side effect associated with the use of MSCs, other short-term adverse events, such as allergic reactions, secondary infections, viral reactivations, and thromboembolic events, should be closely monitored and promptly managed. On the other hand, cryopreserved MSC products usually contain dimethyl sulfoxide, which may cause acute infusion toxicities, such as headache, dizziness, nausea, vomiting, and allergic reactions. Premedication with antihistamines can alleviate these symptoms. Meanwhile, future studies are needed to assess possible long-term complications, such as ectopic tissue formation and tumorigenesis.

## 7. Conclusions

**Figure 4:**
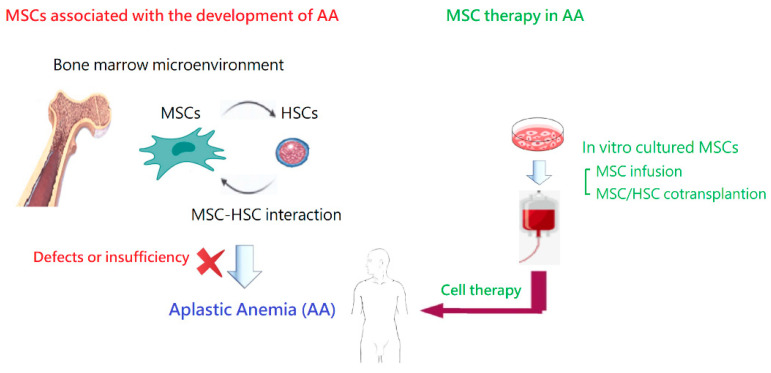
Association between MSCs and the development of AA and MSC therapy in AA.

Acquired idiopathic AA is a rare but life-threatening bone marrow failure syndrome, with a complex pathophysiology. In this review article, we summarized what is known today regarding the association between MSCs in the pathogenesis of the disease and the use of MSCs for patients with AA (Figure 4). On the whole, MSCs are promising for the management of AA, especially when co-transplanted during HSCT. Meanwhile, there are several important issues regarding their utility in AA and other clinical situations. A variety of challenges still lay ahead! With deeper knowledge from basic studies and clinical applications, we anticipate that more patients can benefit from the therapeutic effects of MSCs, including those with AA. While no severe short-term adverse effects have been observed, long-term safety still needs to be assessed in future studies.

## Figures and Tables

**Table 1:** MSC transplantation in animal models of AA.

Reference	Animals	Attempt to Induce AA	MSC Source	MSC Administration	Efficacy of MSC Therapy
[111]	BALB/c mice	IR (5.5 Gy)	Allogenic BM	2.5 × 10^7^/kg, IV, once, 4 h after IR	Rapid recovery of blood cells; lower apoptotic ratio of BM cells; increase in BM hematopoietic islands; recovery of CFU-GM and CFU-F
[111]	BALB/c mice	IR (8 Gy)	Allogenic BM	Three groups: 2.5, 5, or 15 × 10^7^/kg, IV, once, cotransplanted with 1 × 10^9^/kg of donor BM cells	Improvements in survival, but not in those receiving 15 × 10^7^/kg MSCs
[112]	BALB/c mice	IR (7 Gy)	Human cord blood	2 × 10^6^, IV, once, 4 h after IR	Greater proliferation of BM and PB cells; better hematopoietic reconstitution
[113]	BALB/c mice	IR (5.8 Gy)	Human umbilical cords + ECSOD	1 × 10^6^, IV, twice, 1 h and 48 h after IR	Improvements in survival; promoted hematopoietic recovery; decrease in radiation-induced O_2_^-^ and apoptosis
[114]	B6D2F1 mice	IR (5 Gy)	Autologous adipose tissue	6 × 10^5^, IV, once, cotransplanted with HSCs	Improvements in hematopoietic reconstitution; enhanced donor HSC engraftment; facilitated migration and homing of donor HSCs
[115]	BALB/cBy mice	IR (4 Gy) + lymph node cell infusion	Allogenic multiplacenta pooled cells	1 × 10^7^/kg, IP, once, 7 days after IR	Longer survival time; improved PB hemoglobin levels, but not BM architecture response
[116]	BALB/c mice	IR (4 Gy)	Rat adipose tissue	2 × 10^6^, IV, once, immediately after IR	Better recovery of platelets and leukocytes in PB, but not RBCs; increase in BM total CFUs and megakaryocyte-CFUs; improved BM cellularity; inhibited apoptosis of BM cells; antiapoptotic effects mediated via the PI3K/Akt pathway
[117]	CB6F1 mice	IR (5 Gy) + lymph node cell infusion	Human gingiva tissue	2 × 10^6^, IV, once, 6 days after IR	Improvements in survival; attenuated T cells-mediated BM damage; protective effects by regulating the balance of Th1, Th17, and Tregs
[100]	CByB6F1 mice	IR (5 Gy) + lymph node cell infusion	Human umbilical cords	1 × 10^6^, IV, once, 3 days after IR	Improvements in survival; alleviate body weight decline; ameliorated pathological damages; regained hematopoiesis and immunoregulatory capacity

AA: aplastic anemia; BM: bone marrow; CFU: colony-forming unit; CFU-F: colony-forming unit-fibroblast; CFU-GM: colony-forming unit-granulocyte macrophage; ECSOD: extracellular superoxide dismutase; HSCs: hematopoietic stem cells; IP: intraperitoneal injection; IR: irradiation; IV: intravenous infusion; MSC: mesenchymal stem cell; PB: peripheral blood; RBCs: red blood cells; Tregs: regulatory T cells.

**Table 2:** Studies on MSC infusion for patients with AA.

Reference	Number of Patients	Disease Status (SAA/NSAA)	MSC Source	MSC Administration	Main Findings
[124]	1	Refractory SAA	Allogeneic relative BM	Twice (2 × 10^6^/kg and 6 × 10^6^/kg), IV	No effects on hematopoiesis; partial recovery of BM stroma; MSC engraftment detected in recipient BM; death due to invasive fungal infection
[125]	18	Refractory AA (4/14)	Allogeneic relative BM	5.0–7.1 × 10^5^/kg, IV, once	Achieved CR or PR at 1 year in 6 patients; no major adverse events; increase in Tregs in PB
[126]	9	Refractory or relapsed AA (7/2)	Allogeneic HLA- mismatched unrelative BM	1.3–4.5 × 10^6^/kg, IV, weekly, 2–5 times	Achieved PR at 6 months in only 2 patients; no significant improvement in clinical hematologic responses; no infusion-related adverse events; four deaths due to heart failure and infections; no evidence for MSC engraftment in recipient BM
[127]	74	Refractory AA (24/50)	Allogeneic BM	1–2 × 10^6^/kg, IV, weekly, for 4 weeks	28.4% overall response (6.8% CR; 21.6% PR); 87.8% overall survival at 2 years; no significant adverse events
[128]	9	Newly diagnosed SAA in children	Allogeneic umbilical cords	1 × 10^6^/kg, IV, weekly, for 3 weeks	Compared to that in patients receiving IST alone, no significant differences in early response rates and long-term outcomes

AA: aplastic anemia; BM: bone marrow; CR: complete response; IST: immunosuppressive therapy; IV: intravenous infusion; MSC: mesenchymal stem cell; NSAA: non-severe aplastic anemia; PB: peripheral blood; PR: partial response; SAA: severe aplastic anemia; Tregs: regulatory T cells.

**Table 3:** Studies on co-transplantation of MSCs and HSCs for patients with SAA.

Reference	Patient Number	Status	MSC Source	MSC Administration	Main Findings
[129]	1	Refractory SAA for MUD PBSCT	Third-party donor	1 × 10^6^/kg, twice, 1st dose on day 0 and 2nd boost on day + 26	Hematopoietic engraftment; sustained remission
[130]	2	Children with refractory SAA for MUD PBSCT	5/6 MUD umbilical cords	4.2–4.3 × 10^6^/kg, once, 4 h before HSC infusion	Faster neutrophil and platelet engraftment; no acute or chronic GVHD; no infusion-related adverse events
[131]	6	Children with refractory SAA for HSCT	Umbilical cords or BM	0.85–2.5 × 10^6^/kg, once, before HSC infusion	Stable HSC engraftment; no severe acute GVHD; no infusion-related adverse events
[132]	37	Pediatric patients with refractory SAA for allogeneic HSCT	Umbilical cords	0.78–3.41 × 10^6^/kg, one (n = 20) or more times (n = 17), infusion frequency depending on GVHD severity	Evidence of proliferative BM; 100% successful HSC engraftment; 74.2% OS; decrease in death due to infection and organ failure; no effective control in patients with severe GVHD and concomitant serious infection; no significant adverse effects
[133]	17	Refractory SAA for haploidentical HSCT	Umbilical cords	2.87–10 × 10^6^/kg, 6 h before HSC infusion	No primary graft failure; 23.5% grade III–IV acute GVHD; 14.2% moderate and severe chronic GVHD; 76.5% OS at 6 months; no infusion-related adverse events
[134]	44	SAA for haploidentical HSCT	Allogeneic BM	3.2–4.1 × 10^6^/kg, 1st dose on day 0 and 2nd dose on day + 14, additional doses weekly for 1–4 weeks if poor graft function and severe GVHD	Reduced graft failure and severe GVHD in haploidentical HSCT: 97.6% hematopoietic reconstitution and full donor chimerism, 29.3% grade II–IV acute GVHD, 14.6% chronic GVHD; 77.3% OS; no infusion toxicity
[135]	24	SAA for haploidentical HSCT	Umbilical cords	5 × 10^5^/kg, once, 4 h before HSC infusion	All achieved donor chimerism within 1 month; 50% acute GVHD; 83.3% OS at 6 months
[136]	33	Children with SAA for haploidentical HSCT	Donor BM	1 × 10^6^/kg, twice, 1st dose on day 0 (6 h before HSC infusion) and 2nd dose on day + 14	Faster hematopoietic implantation; 100% hematopoietic reconstitution and full donor chimerism; effective prevention of severe GVHD (25.71% grade II–IV acute GVHD, 22.86% chronic GVHD); 85.71% OS; no infusion toxicity
[137]	25	SAA for haploidentical HSCT	Umbilical cords	1 × 10^6^/kg, once, on day 0	All achieved neutrophil engraftment; achieved platelet engraftment in 23 patients; 32% grade II acute GVHD; no grade III–IV acute GVHD; 28% chronic GVHD; 71.78% OS
[138]	47	SAA for haploidentical HSCT	Umbilical cords	1 × 10^6^/kg, once, 4 h before HSC infusion	Compared to those without MSC infusion, faster neutrophil engraftment, lower cumulative incidence of chronic GVHD, similar rate of acute GVHD, better 5-year OS

BM: bone marrow; GVHD: graft-versus-host disease; HSC: hematopoietic stem cell; HSCT: hematopoietic stem cell transplantation; MSC: mesenchymal stem cell; MUD: matched unrelated donor; OS: overall survival; PBSCT: peripheral blood stem cell transplantation; SAA: severe aplastic anemia.

## Data Availability

Not applicable.
